# Bioactive Properties and Phenolic Compound Profiles of Turnip-Rooted, Plain-Leafed and Curly-Leafed Parsley Cultivars

**DOI:** 10.3390/molecules25235606

**Published:** 2020-11-28

**Authors:** Ângela Liberal, Ângela Fernandes, Nikolaos Polyzos, Spyridon A. Petropoulos, Maria Inês Dias, José Pinela, Jovana Petrović, Marina Soković, Isabel C.F.R. Ferreira, Lillian Barros

**Affiliations:** 1 Mountain Research Center (CIMO), Institute Polytechnic of Bragança, Campus de Santa Apolónia, 5300-253 Bragança, Portugal; angela.liberal@ipb.pt (Â.L.); afeitor@ipb.pt (Â.F.); maria.ines@ipb.pt (M.I.D.); jpinela@ipb.pt (J.P.); iferreira@ipb.pt (I.C.F.R.F.); 2 Department of Agriculture Crop Production and Rural Environment, University of Thessaly, Fytokou Street, 38446 Volos, Greece; npolyzos@uth.gr; 3 Institute for Biological Research “Siniša Stanković”-National Institute of Republic of Serbia, University of Belgrade, Bulevar despota Stefana 142, 11000 Belgrade, Serbia; jovana0303@ibiss.bg.ac.rs (J.P.); mris@ibiss.bg.ac.rs (M.S.)

**Keywords:** *Petroselium crispum*, phenolic compounds, antioxidant activity, antimicrobial activity, apigenin, turnip-rooted parsley

## Abstract

*Petroselinum crispum* Mill., Fuss., is a culinary vegetable used as an aromatic herb that garnishes and flavours a great variety of dishes. In the present study, the chemical profiles and bioactivities of leaf samples from 25 cultivars (three types: plain- and curly-leafed and turnip-rooted) from this species were assessed. Seven phenolic compounds were identified in all the varieties, including apigenin and kaempherol derivates. Apigenin-*O*-pentoside-*O*-hexoside was the major compound in all the tested parsley types (20, 22 and 13 mg/g of extract, respectively) and responsible for its excellent antioxidant activity, also investigated in this study. Antimicrobial activities were also explored, and the results revealed a good bioactivity against specific tested pathogens, such as bacteria and fungi. In conclusion, the leaves of all the types of *P. crispum* are a good source of natural bioactive compounds that confer health benefits, and thus, they should be part of a balanced and diversified diet.

## 1. Introduction

Aromatic herbs, grown and consumed for hundreds of years, are gaining popularity due to their ability to enhance and complement the flavour and aroma in a wide range of foods and due to their composition in terms of essential macro- and micronutrients such as vitamins, minerals and other bioactive compounds [1]. Parsley (*Petroselium crispum* (Mill.) Fuss.), commonly known as English or garden parsley, allegedly native to Sardinia (Mediterranean region), is a species of the *Apiaceae* or *Umbelliferae* family and is a popular culinary vegetable grown worldwide and extensively used as a flavouring and aromatic food additive [2].

Besides its widespread use as a fresh legume and garnishment, *P. crispum* is proposed for diverse therapeutic determinations in daily medicine for antioxidant, hepatoprotective, anti-diabetic, antibacterial, antifungal, analgesic, diuretic, hypotensive, gastroprotective, immunosuppressant and other effects [3], which are attributed to a broad variety of active compounds detected in this plant. Parsley plant parts (the leaf, stem and root) are rich sources of bioactive compounds such as furanocoumarins (e.g., xanthoxin, trioxalen and angelicin), essentials oils (e.g., sesquiterpene hydrocarbons, monoterpene hydrocarbons and alcohols, furanocoumarins, aldehydes and aromatic compounds), flavonoids (e.g., quercetin, apiol, myristicin, apigenin, luteolin and their glycosides), carotenoids (e.g., neoxanthin, β-carotene, lutein and violaxanthin), vitamins (e.g., tocopherols and A, C and B complexes), minerals (e.g., iron, zinc, calcium and phosphorous) and fatty acids (e.g., linolenic and palmitic acid) [1,3,4,5,6,7,8,9]. Notably, numerous bioactive properties have been attributed to the leaves of *P. crispum*, which are reported for the handling of immune diseases, inflammation, anaemia, hyperlipidaemia and diabetes and in the relief of the symptoms of allergies, chronic bronchitis, dyspepsia, hypotension, thrombosis and strokes, among other diseases [5,6]. On the other hand, the aerial portions of the plant can also be used as an abortifacient [10]. According to the literature, the aqueous and organic solvent extracts of parsley leaves contain various phytochemicals such as furanocoumarins that may exhibit phytotoxic effects [11], while Ancuceanu et al. [12] suggested a significant variation in polyphenols, flavones and iron among plant organs (roots, stems and leaves). Dadan et al. [4] suggested that parsley leaves are a rich source of lutein, whose content may be affected by drying methods and processing pre-treatments. Moreover, Slighoua et al. [13] detected ferulic and gallic acid and quercetin in hydroethanolic extracts of parsley aerial parts, while Misi et al. [13] suggested sedanolide as the major compound in parsley seed extracts obtained via supercritical fluid extraction. According to Aissani et al. [14], the methanolic extracts contained more phenolic acids and total phenolic compounds than aqueous extracts, and they suggested quinic acid, gallic acid, acacetin, protocatechuic acid and cirsilineol as the major compounds.

Plain-leafed (*P. crispum* spp. *neapolitanum*), curly-leafed (*P. crispum* spp. *crispum*) and turnip-rooted (*P. crispum* ssp. *tuberosum*) are the three main types of parsley, and various cultivars of each are cultivated worldwide [15]. According to the literature, the leaves of the turnip-rooted type can similarly be explored in relation to different bioactivities, since they also have aromas and flavours like the cultivars of leafy types of parsley [8,16,17]. Some disparities are noted between cultivars, and phenotypic diversity is dominant in characteristics such as morphology, growing routine, blossom colour, stems, leaves and chemical configuration [18]. In this line, the unstable nature of abiotic factors throughout plant vegetation adjusts the specificities of metabolic courses and the chemical profile and composition of biologically active constituents. The biosynthetic processes may be influenced by numerous factors such as the meteorological conditions, the harvesting time, the genotype, the irrigation regime and the planting density, among others [19,20,21,22]. It was also observed that parsley in the autumn harvest accumulates more polyphenols and pigment complexes (β-carotene) than plants grown in spring, which could be associated with the variation of the environmental effects. Moreover, Rowland et al. [23] and Najla et al. [24] suggested that deficit irrigation may increase vitamin C and anthocyanin contents in parsley leaves.

Therefore, in the present work, it was intended to examine the diversification of parsley crops by assessing the phenolic profiles and bioactive compound contents in the leaves of 25 cultivars belonging to the three types (namely, plain-leafed, curly-leafed and turnip-rooted type), grown in central Greece.

## 2. Results and Discussion

### 2.1. Phenolic Compound Composition

Information about the phenolic compounds identified in *Petroselinum crispum* leaves are presented in Table 1, namely, the retention time, λmax, pseudomolecular ions, major fragment ions in MS^2^, and tentative identification of each individual compound. Our study revealed the presence of seven compounds, three being apigenin derivatives, tentatively recognized as apigenin-*O*-pentoside-*O*-hexoside (peak 1; λmax, 336 nm; [M − H]—at *m/z* 563, [25]) and apigenin-*O*-acetyl-hexosyl-pentoside isomers 1 and 2 (peaks 4 and 5; λmax, ~340 nm; [M − H]—at *m/z* 605, respectively, [25]). Additionally, four kaempherol derivatives were also detected and tentatively recognized as kaempherol-3-*O*-rutinoside (peak 2; λmax, 342 nm; [M − H]—at *m/z* 593, positively identified using a commercial standard, [26]), kaempherol-*O*-deoxyhexosyl-hexoside (peak 3; λmax, 343 nm; [M − H]—at m/z 593, [26]) and kaempferol-(*p*-coumaroyl)hexoside isomers 1 and 2 (peaks 6 and 7; λmax, ~343 nm; [M − H]—at *m/z* 635, respectively, [26]). The standard compound used to positively identify peak 2 as kaempherol-3-*O*-rutinoside was previously described and quantified with the corresponding exact compound by Pires et al. [26].

Likewise, El-Zaeddi et al. [20] and Slimestad et al. [27] identified various flavonoid glucosides (apigenin, isorhamnetin, diosmetin and their derivatives), whereas no other classes of polyphenols were detected. The presence of apigenin and kaempherol derivates was also reported in *P. crispum* leaf aqueous extracts by Epifanio et al. [28]. However, these authors also found more phenolic compounds compared to our study, namely, luteolin, chrysoeriol, quercetin and isorhamnetin derivatives, adding a total of 30 compounds. Moreover, Chaves et al. [29] also identified flavonoids such as apigenin, cosmosiin, apiin and coumarin as the major compounds detected in aqueous extracts of plain-leafed parsley leaves. By contrast, Nour et al. [30] suggested myricetin and quercetin as the major phenolic compounds in parsley leaves, and they also detected several phenolic acids in considerable amounts (salicylic, sinapic, ferulic and ellagic acid), whereas Justesen and Knuthsen [31] detected apigenin, quercetin and luteolin. According to Mazzucotelli et al. [32], catechin was the most abundant free phenolic compound, whereas p-cumaric acid and rutin were identified as bound polyphenols. Similarly, Derouich et al. [32] identified chlorogenic, p-coumaric and caffeic acid as the major polyphenols in hydromethanolic extracts of parsley leaves. Therefore, the higher numbers of compounds detected in other reports may be related to the implemented extraction methods as well as to other factors already reported in the literature (genotype, growing conditions, growth substrate, harvesting time, cultivation practices etc.), which can alter the chemical composition and the profile of secondary metabolites in plants [7,12,19,20,33,34,35,36]. Moreover, Grúz et al. [37], who detected hydroxycinnamic acids such as coumaric, ferulic and sinapic acids in parsley leaves, suggested that these compounds are prone to oxidation and degradation, which may affect their identification in plant samples.

Concerning the quantification of the phenolic compounds, whose results are presented in Table 2, the major compound was apigenin-*O*-pentoside-*O*-hexoside (20, 22 and 13 mg/g of extract in the plain-leafed, curly-leafed and turnip-rooted types, correspondingly). In particular, the uppermost tenor of the major and whole flavonoids was detected in Rialto Bejo (plain-leafed type), Mooskrause (curly-leafed type) and Alba (turnip-rooted type), indicating a great diversity among the tested genotypes. However, when considering the overall means of each parsley type, significant differences were recorded only in individual compounds and not in total phenolic compound content. Similarly to our study, Jadczak et al. [38] reported significant differences in total polyphenol content among various cultivars of leafy type parsley. In general, apigenin derivatives were the main compounds found in *P. crispum* in other reports such as the study of Pápay et al. [39], who similarly considered that apigenin and its glycosides were, in general, the most abundant phenolics present in parsley. According to Patel et al. [40], apigenin is a potent chemopreventive agent that drives multiple pathways in cancer prevention and therapy, exhibiting good antiproliferative activity and properties that promote cell-cycle arrest and apoptosis. Furthermore, apigenin derivates can also prevent oxidation, regulate the host immune system and control cell signalling. Likewise, kaempherol derivatives play a pivotal role in human health, with many studies reporting their antioxidant, cardioprotective, anticancer, anti-inflammatory and neuroprotective benefits [41]. Our investigation showed that the total phenolic compound content in the hydroethanolic extract was 29 mg/g of extract (plain-leafed), 32 mg/g of extract (curly-leafed) and 38 mg/g of extract (turnip-rooted), showing no significant differences (*p* > 0.05) among the tested types. In contrast to our study, Dobričević et al. [7] suggested that apart from flavonoids, the ethanolic extracts of the aerial parts of various types of cultivars (three turnip-rooted, one plain-leafed and two curly-leafed cultivars) also contained a non-flavonoid polyphenolic fraction in amounts that differed among the studied cultivars. Similarly, Nour et al. [30] reported that although flavonoids comprise the highest portion (260.55 mg of quercetin equivalents/100 g of extract) of total phenolic compounds (360.89 mg of gallic acid equivalents/100 g of extract), a significant non-flavonoid content was also detected (27.8% or 100.34 mg/100 g of extract). The differences in phenolic compound composition and individual compound contents could be partially attributed to different extraction protocols and extraction pre-treatments, since molecules of different polarity may be identified by using different solvents [14,42,43,44]. 

### 2.2. Antioxidant Activity

The outcomes obtained in the Thiobarbituric acid reactive substances (TBARS) and oxidative hemolysis inhibition (OxHLIA) assays are shown in Table 3. Regarding the TBARS assay, all three types presented excellent antioxidant activities, with EC50 values ranging from 1.5 to 1.6 mg/mL, showing no significant differences (*p* > 0.05) among them. On the other hand, regarding the OxHLIA assay, the curly-leafed type showed lower antihaemolytic activity (IC50 value of 366 µg/mL at Δt = 60 min) than the other two types, whereas the turnip-rooted type showed the strongest activity at Δt = 60 min (IC50 value of 118 µg/mL).

Regarding the differences between the cultivars of the same type, Fesival 68, Mooskrause and Vistula exhibited the highest activities as determined by the TBARS assay for the plain-leafed, curly-leafed and turnip-rooted types, correspondingly. Similarly, in the OxHLIA assay, the Fest (plain-leafed) and Moss Curled 2 (curly-leafed) cultivars were the most effective for both Δt = 60 min and 90 min. In the case of the turnip-rooted cultivars, Linga, Halblange Berlinska, Vistula and Lenka were the most effective at Δt = 60 min, while Linga, Halblange Berlinska, Vistula and Kaśka were similarly effective at Δt = 90 min.

Several studies reported the antioxidant activity of *P. crispum* leaves. For example, Nielsen et al. [42] carried out an experiment with 14 people where leaves of curly-leafed parsley were added to their daily diet for one week (fresh or included in meals). The results of this study demonstrated an intensification of antioxidant enzymes related to the levels of the group that received the basic diet (control). For these authors, apigenin was the major compound responsible for these results, which is also in agreement with our study, considering the high levels of apigenin derivatives being found in the species under study. The antioxidant activity of a *P. crispum* aqueous extract was also studied by Epifanio et al. [28] through the 2,2-diphenyl-1-picrylhydrazyl (DPPH) radical scavenging activity assay, which also revealed EC50 values of 15.5 mg/mL, concordant with the strong antioxidant activity of this species. Moreover, in the same study, it was suggested that apiin and apigenin exhibit strong protection against lipid peroxidation as determined via the TBARS assay, although no synergistic effects were observed for the joint incubation of these compounds [28]. Although phenolic compound content was associated with antioxidant activity in several studies [5,30,42,45], our results did not confirm this trend, probably due to different assays being performed and the presence of other compounds not determined in this study.

### 2.3. Antimicrobial Activity

The antimicrobial properties of the three tested types of *P. crispum* leaves are shown in Table 4. According to the results, the highest activity was recorded against *Escherichia coli*; the extracts from several cultivars belonging to the plain-leafed and the turnip-rooted types showed similar minimal inhibition concentration (MIC) and minimal bactericidal concentration (MBC) values to one of the positive controls (E224). Moreover, the majority of the plain-leafed cultivars (except for Rialto Bejo) showed high antibacterial activities against *Staphylococcus aureus*, with MIC values comparable to those for the positive control E224. Similar results were recorded for the turnip-rooted cultivars Olomuńcka, Pólna, Lenka, Kaśka, Cucrowa and Alba. For *Bacillus cereus* and *Salmonela Tymphimurium*, a varied response was observed, and only specific cultivars belonging to the three tested types exhibited similar MIC values to the positive control E224. By contrast, all the tested extracts presented lower activities against *Listeria monocytogenes* and *Enterobacter cloacae* compared to the used positive controls, especially E224.

The antifungal activities of the leaves’ extracts are shown in Table 5. All the extracts presented antifungal activity with different MIC and MFC totals against different fungi.

The antibacterial activities of parsley against various strains have also already been reported for the essential oils of the species [41,46], while ethanolic extracts of the leaves and seeds showed efficacy against *S. typhi*, *S. aureus* and *Klebsiella pneumonia* [47,48]. Moreover, the antifungal assets of *P. crispum* have previously been described by Abdu and Hauwa [47], who also documented effectiveness against *Mucor* species, *Aspergillus flavus* and *Candida albicans*, thus confirming its antimicrobial efficiency against a wide range of microorganisms.

The most interesting results were observed against *Aspergillus fumigatus* and *Penicillium verrucosum* var. *cyclopium*, where several cultivars showed higher effectiveness compared to the tested positive controls (E211 and E224). Moreover, the MIC values of various leaves’ extracts were higher than those of the positive controls when tested against *A. ochraceus*, *A. versicolor* and *Trichoderma viride*, although no differences were observed in the minimum fungicidal concentration (MFC) values between the plant extracts and the positive controls for specific cultivars. Finally, plant extracts of specific cultivars exhibited similar MIC values to the positive controls against *P. funicolosum*, whereas the MFC values were lower than the controls’.

## 3. Materials and Methods

### 3.1. Growing Conditions

The seeds of twenty-five different cultivars of parsley (*Petroselinum crispum* (Mill.) Fuss.) were directly sown in November 2018 in 6 L plastic containers with peat (Klassman-Deilmann KTS2, Geeste, Germany) and perlite (2:1; *v/v*). A list with the names and the types of cultivars is presented in Table 6. After emergence, young seedlings were thinned to three plants per container with equal distances, using 15 containers for each cultivar [49]. Cultivation was initially performed in an unheated glasshouse at the experimental farm of the University of Thessaly in Velestino, Greece, while the containers were moved outdoors in March 2019, given the rising temperatures within the greenhouse. Throughout cultivation, irrigation was performed once or twice per week, depending on the conditions, via a sprinkler irrigation system, while the plants were also fertigated manually twice a month with a nutrient solution comprising 200 mg/L of N–P–K (Atlas 20-20-20 + TE; Gavriel S.A., Volos, Greece) [50,51] in amounts ranging between 150 and 300 mL per container, depending on the plant growth and the environmental circumstances.

On each occasion, all the plants received the same amount of nutrient solution. Pest control was carried out according to the best practice guide recommended for parsley, whereas no weed control was needed due to soilless cultivation. The plants were harvested in June 2019, cutting the mature leaves from the base of the plant, near the substrate. Fresh leaves from all the containers from each cultivar were compiled in batch samples, put in vacuum-wrapped plastic bags and stored at −80 °C until lyophilization and grinding to a fine powder.

### 3.2. Extract Preparation

Hydroalcoholic extractions were performed by stirring the leaf material; briefly, 1 g of lyophilized sample was twice subjected to a 1 h extraction (25 °C at 150 rpm) with 30 mL of ethanol (Sigma-Aldrich, St. Louis, MO, USA)/water (80:20; *v/v*) and then filtered through Whatman No. 4 paper. After that, the ethanol was removed using a rotary evaporator (Büchi R-210, Flawil, Switzerland), and the samples were frozen and lyophilized (FreeZone 4.5 model 7750031, Labconco, Kansas City, MO, USA) for further analysis [52].

### 3.3. Phenolic Compound Composition

Phenolic compounds were assessed in the lyophilized hydroethanolic extracts prepared as described above and redissolved in ethanol/water (80:20; *v/v*) to a final concentration of 10 mg/mL. Evaluation was carried out using a Dionex Ultimate 3000 UPLC (Thermo Scientific, San Jose, CA, USA), and detection was performed with a diode array detector (DAD) coupled to an electrospray ionization mass spectrometry detector (ESI/MS) (API 3200 Qtrap, Applied Biosystems, Darmstadt, Germany). The operating conditions were previously described in detail by Bessada et al. [52], as were the identification and quantification procedures. The results are given as mg per g of extract.

### 3.4. Bioactive Properties

#### 3.4.1. Antioxidant Activity

The antioxidant activity of the three types of *P. crispum* was evaluated in hydroethanolic extracts through two cell-based assays: the oxidative haemolysis inhibition (OxHLIA) and the thiobarbituric acid reactive substances (TBARS) assays, according to the procedure reported in detail by Lockowandt et al. [53]. The antihaemolytic activity was determined through the OxHLIA assay, and the results are expressed as IC_50_ values, which is the extract concentration (µg/mL) required to prevent the oxidative haemolysis of 50% of the erythrocytes for ∆t of 60 and 120 min. For the TBARS assay, lipid peroxidation inhibition was evaluated through the colour concentration of malondialdehyde–thiobarbituric acid (MDA-TBA) and measuring its absorbance at 532 nm. The inhibition ratio (%) was calculated using the following formula: [(A − B)/A] × 100%, where A and B were the absorbance values of the control and the sample solutions, respectively [53]. The results are presented as EC_50_ values (mg/mL), which reflect the sample’s concentrations that provide 50% antioxidant activity. Trolox (Sigma-Aldrich, St. Louis, MO, USA) was the positive control used.

#### 3.4.2. Antimicrobial Activity

The antimicrobial and antifungal activities were assessed through a microdilution method [54]. The antibacterial properties were tested against the Gram-positive bacteria *Staphylococcus aureus* (ATCC 6538), *Bacillus cereus* (food isolate) and *Listeria monocytogenes* (NCTC 7973), as well as the following Gram-negative bacteria: *Escherichia coli* (ATCC 25922), *Salmonella enterica* sp. Typhimurium (ATCC 13311) and *Enterobacter cloacae* (ATCC 35030). For the antifungal assays, six micromycetes were used: *Aspergillus fumigatus* (ATCC 9197), *A. niger* (ATCC 6275), *A. versicolor* (ATCC 11730), *Penicillium funiculosum* (ATCC 36839), *Trichoderma viride* (IAM 5061) and *P. verrucosum* var. *cyclopium* (food isolate). In particular, *Bacillus cereus* and *Penicillium verrucosum* var. *cyclopium* strains were isolated from cream cheese, as published before [53]. The minimum inhibitory and bactericidal concentrations (MICs and MBCs) were determined using the serial dilution technique in 96-well microtiter plates (ThermoFisher Scientific, Lisbon, Portugal). The bacterial suspension was adjusted with sterile saline to a concentration of 1.0 × 10^5^ CFU/mL. The MICs obtained from testing the susceptibility of various bacteria to the tested extracts were determined by a colorimetric microbial viability assay based on the reduction of INT (p-iodonitrotetrazolium violet (syn, 2-(4-iodophenyl)-3-(4-ni-trophenyl)-5-phenyltetrazolium chloride) Sigma) and compared with the positive control for each bacterial strain. The MBCs were determined by the reinoculation of 10 µL of medium with inoculum and the tested extracts in fresh clean medium and further incubation for 24 h at 37 °C. Subsequently, the lowest concentrations without visible microbial growth were defined as the MBCs, indicating 99.5% death of the bacterial strain. Similarly, the minimum inhibitory and fungicidal concentrations (MICs and MFCs) were obtained using the serial dilution technique in 96-well microtiter plates. The fungal spores were washed from the surface of the agar plates with sterile 0.85% saline containing 0.1% Tween 80 (*v/v*). The spore suspension was adjusted with sterile saline to a concentration of approximately 1.0 × 10^5^ in a final volume of 100 µL per well. The inocula were stored at 4 °C for further use. Dilutions of the inocula were cultured on solid malt agar (Sigma-Aldrich, St. Louis, MO, USA) to verify the absence of contamination and to check the validity of the inocula. The tested extracts were added to malt extract broth, after which the appropriate concentrations of the fungal inocula were added. The lowest concentrations without visible growth (under a binocular microscope) were defined as the MICs. The fungicidal concentrations (MFCs) were determined by the reinoculation of 10 µL of medium with inoculum and tested extracts in microtiter plates containing 100 µL of fresh broth per well and further incubation for 72 h at 25 °C. The lowest concentration with no visible growth was defined as the MFC, indicating the 99.5% killing of the original inoculum. E211 and E224 (Sigma-Aldrich, St Louis, MO, USA) were used as positive controls.

### 3.5. Statistical Analysis

The experiment was performed according to a completely randomized design (RCD) with 15 repetitions (pots) per cultivar. The results for the chemical composition and bioactive properties are presented as means ± SD (n= 3), while all the assays were performed in triplicate. The statistical analysis was performed by using the SPSS v. 23.0 software for Windows (IBM Corp., Armonk, NY, USA) and using the one-way analysis of variance (ANOVA), while the comparison of means was carried out with Tukey’s HSD test (*p* < 0.05) when statistically significant differences were detected.

## 4. Conclusions

Our results suggest that *P. crispum* leaves are an excellent source of bioactive compounds such as the various detected polyphenols. Their composition, rich in apigenin and kaempherol derivatives, as already reported in several other studies, is indicated as being responsible for their high antioxidant capacity and could also be associated with the observed antimicrobial properties for a variety of microorganisms such as bacteria and fungi. Thus, given *P. crispum*’s rich composition in valuable bioactive compounds, the further valorisation of this species in the human diet should be targeted, either through its consumption as a garnish or by its incorporation into less-conventional foods, representing a scientific development in suggesting a balanced and diversified diet, rich in bioactive compounds. Moreover, to the best of our knowledge, our study reports comparative results regarding the phenolic compound profiles and the bioactive properties of several parsley cultivars belonging to all three types, namely, plain- and curly-leafed and turnip-rooted parsley, suggesting great compositional variability within the parsley germplasm.

## Figures and Tables

**Table 1:** Retention times (Rt), wavelengths of maximum absorption in the visible region (λmax), mass spectral data and tentative identification of the phenolic compounds present in the hydroethanolic extracts of the tested parsley leaves.

Peak	Rt (min)	*λ*_max_ (nm)	[M − H]^−^ (*m/z*)	MS^2^ (*m/z*)	Tentative Identification
**1**	21.08	336	563	431(21), 269(100)	Apigenin-*O*-pentoside-*O*-hexoside
**2**	22.49	342	593	285(100)	Kaempherol-3-*O*-rutinoside
**3**	23.58	343	593	285(100)	Kaempherol-*O*-deoxyhexosyl-hexoside
**4**	24.47	340	605	563(100), 269(51)	Apigenin-*O*-acetyl-hexosyl-pentoside isomer 1
**5**	26.14	338	605	563(100), 269(48)	Apigenin-*O*-acetyl-hexosyl-pentoside isomer 2
**6**	27.06	342	635	593(55), 285(100)	Kaempferol-(*p*-coumaroyl)hexoside isomer 1
**7**	27.77	343	635	593(57), 285(100)	Kaempferol-(*p*-coumaroyl)hexoside isomer 2

**Table 2:** Quantification (mg/g of extract) of the phenolic compounds present in the hydroethanolic extracts of the tested parsley leaves (mean ± SD, *n* = 3).

Cultivar Type	Cultivar Name	Peak 1	Peak 2	Peak 3	Peak 4	Peak 5	Peak 6	Peak 7	Σ Flavonoids
Plain-leafed		0.8 ± 0.4 ^B^	1.3 ± 0.5 ^B^	1.1 ± 0.6 ^B^	5 ± 1 ^C^	0.22 ± 0.09 ^B^	0.3 ± 0.1 ^B^	29 ± 7 ^A^	20 ± 6 ^A^
	Festival 68	16.1 ± 0.4 ^d^	0.92 ± 0.05 ^b^	0.92 ± 0.05 ^c^	0.49 ± 0.02 ^e^	3.94 ± 0.02 ^c^	0.225 ± 0.007 ^b^	0.125 ± 0.003 ^c^	22.7 ± 0.4 ^c^
	Astra	19.5 ± 0.3 ^c^	0.48 ± 0.02 ^c^	0.97 ± 0.04 ^c^	0.92 ± 0.02 ^c^	6.6 ± 0.2 ^a^	0.211 ± 0.001 ^b^	0.36 ± 0.01 ^a^	29.1 ± 06 ^b^
	Gigante Di Italia	14.1 ± 0.2 ^e^	0.29 ± 0.02 ^d^	0.750 ± 0.00 ^d^	0.79 ± 0.06 ^d^	5.2 ± 0.3 ^b^	0.118 ± 0.006 ^d^	0.205 ± 0.002 ^b^	21.4 ± 0.4 ^d^
	Fest	21.3 ± 0.3 ^b^	0.939 ± 0.004 ^b^	2.0 ± 0.1 ^a^	1.21 ± 0.06 ^b^	3.23 ± 0.01 ^d^	0.162 ± 0.001 ^c^	0.37 ± 0.02 ^a^	29.2 ± 0.4 ^b^
	Rialto Bejo	30.3 ± 0.2 ^a^	1.26 ± 0.01 ^a^	1.74 ± 0.03 ^b^	2.14 ± 0.04 ^a^	5.2 ± 0.1 ^b^	0.37 ± 0.02 ^a^	0.38 ± 0.02 ^a^	41.3 ± 0.3 ^a^
Curly-leafed		22 ± 2 ^A^	1.5 ± 0.9 ^A^	0.7 ± 0.1 ^C^	1.2 ± 0.2 ^A B^	6 ± 6 ^A B^	0.6 ± 0.5 ^A^	0.11 ± 0.04 ^B^	32 ± 4 ^A^
	Depuis 1743	24.0 ± 0.1 ^a^	0.362 ± 0.008 ^c^	0.566 ± 0.007 ^c^	1.04 ± 0.01 ^c^	5.8 ± 0.2 ^b^	0.094 ± 0.006 ^c^	0.059 ± 0.005 ^c^	31.9 ± 0.4 ^b^
	Moss Curled 2	19.27 ± 0.04 ^c^	1.58 ± 0.01 ^b^	0.82 ± 0.02 ^a^	1.14 ± 0.09 ^b^	4.0 ± 0.2 ^c^	0.42 ± 0.02 ^b^	0.101 ± 0.002 ^b^	27.3 ± 0.3 ^c^
	Mooskrause	23.6 ± 0.3 ^b^	2.59 ± 0.03 ^a^	0.69 ± 0.03 ^b^	1.49 ± 0.03 ^a^	7.08 ± 0.04 ^a^	1.14 ± 0.02 ^a^	0.163 ± 0.002 ^a^	36.8 ± 0.5 ^a^
Turnip-rooted		13 ± 4 ^B^	0.7 ± 0.4 ^B^	2.2 ± 0.6 ^A^	1.8 ± 0.9 ^A^	8 ± 3 ^A^	0.8 ± 0.4 ^A^	1.9 ± 0.7 ^A^	28 ± 8 ^A^
	Olomuńcka	20.1 ± 0.4 ^a^	1.66 ± 0.07 ^a^	2.0 ± 0.2 ^e f^	1.6 ± 0.1 ^e^	7.3 ± 0.3 ^f^	1.00 ± 0.04 ^c^	0.90 ± 0.06 ^h^	35 ± 1 ^d^
	Pólna	11.5 ± 0.5 ^f^	0.39 ± 0.01 ^j^	2.4 ± 0.1 ^c d^	1.9 ± 0.1 ^d^	11.4 ± 0.6 ^d^	0.89 ± 0.05 ^d e^	2.66 ± 0.03 ^b^	31 ± 1 ^e^
	Linga	15.1 ± 0.3 ^c^	0.76 ± 0.03 ^e^	3.2 ± 0.1 ^a^	1.86 ± 0.01 ^d^	5.7 ± 0.2 ^h i^	0.71 ± 0.05 ^g h^	1.61 ± 0.03 ^e^	28.8 ± 0.5 ^f^
	Halblange Berlinska	11.4 ± 0.2 ^f^	0.49 ± 0.04 ^h^	1.9 ± 0.2 ^f g^	1.02 ± 0.05 ^h i^	5.4 ± 0.3 ^h i^	0.502 ± 0.004 ^j^	1.3 ± 0.1 ^f^	22.0 ± 0.4 ^h^
	Osborne	11.8 ± 0.3 ^f^	0.58 ± 0.05 ^g^	2.8 ± 0.2 ^b^	1.36 ± 0.09 ^f g^	6.19 ± 0.01 ^g h^	0.621 ± 0.001 ^i^	1.67 ± 0.04 ^e^	25.0 ± 0.3 ^g^
	Lenka	9.7 ± 0.5 ^h^	0.300 ± 0.001 ^k^	1.74 ± 0.06 ^g^	0.90 ± 0.04 ^i^	8.7 ± 0.1 ^e^	0.839 ± 0.007 ^e f^	2.1 ± 0.1 ^c^	24.3 ± 0.2 ^g^
	Sonata	11.3 ± 0.3 ^f^	0.44 ± 0.02 ^i j^	2.2 ± 0.1 ^d e^	1.29 ± 0.04 ^g^	6.5 ± 0.2 ^f g^	0.59 ± 0.04 ^i^	1.64 ± 0.02 ^e^	24.0 ± 0.1 ^g^
	Kaśka	14.1 ± 0.4 ^d^	0.567 ± 0.003 ^g^	2.84 ± 0.05 ^b^	1.56 ± 0.01 ^e^	6.7 ± 0.2 ^f^ ^g^	0.66 ± 0.05 ^h i^	2.24 ± 0.06 ^c^	28.7 ± 0.8 ^f^
	Vistula	12.5 ± 0.3 ^e^	0.28 ± 0.02 ^k^	2.5 ± 0.1 ^c^	0.64 ± 0.02 ^j^	4.1 ± 0.3 ^j^	0.23 ± 0.01 ^k^	0.97 ± 0.03 ^g h^	21.2 ± 0.8 ^h^
	Konika	9.8 ± 0.2 ^h^	0.46 ± 0.01 ^h i^	1.34 ± 0.06 ^h^	1.13 ± 0.04 ^h^	7.0 ± 0.1 ^f g^	0.75 ± 0.04 ^f g^	1.57 ± 0.01 ^e^	22.1 ± 0.5 ^h^
	Hanacka	14.0 ± 0.3 ^d^	0.67 ± 0.01 ^f^	2.04 ± 0.04 ^e f^	2.34 ± 0.09 ^c^	7.0 ± 0.1 ^f^	0.82 ± 0.04 ^e f^	1.84 ± 0.07 ^d^	28.7 ± 0.3 ^f^
	Halblange Eagle	11.3 ± 0.2 ^f^	0.77 ± 0.02 ^e^	2.10 ± 0.01 ^e^	2.2 ± 0.1 ^c^	8.9 ± 0.4 ^e^	0.93 ± 0.04 ^c d^	2.2 ± 0.2 ^c^	28.5 ± 0.4 ^f^
	Cukrowa	10.6 ± 0.1 ^g^	0.66 ± 0.02 ^f^	1.74 ± 0.03 ^g^	1.50 ± 0.05 ^e f^	5.25 ± 0.01 ^i^	0.63 ± 0.02 ^h i^	1.56 ± 0.09 ^e^	21.9 ± 0.2 ^h^
	Alba	19.5 ± 0.4 ^a^	1.12 ± 0.01 ^c^	3.26 ± 0.09 ^a^	3.65 ± 0.01 ^a^	13.7 ± 0.8 ^b^	1.64 ± 0.01 ^a^	3.16 ± 0.01 ^a^	46 ± 1 ^a^
	Arat	4.6 ± 0.3 ^i^	0.22 ± 0.01 ^l^	0.62 ± 0.01 ^i^	0.95 ± 0.03 ^i^	4.24 ± 0.02 ^j^	0.31 ± 0.01 ^k^	1.11 ± 0.02 ^g^	12.0 ± 0.3 ^i^

Different capital Latin letters in the same column indicate significant differences between the means of the three types of cultivars according to Tukey’s HSD test at *p* = 0.05, while different small Latin letters in the same column indicate significant differences between the means of the cultivars of the same type according to Tukey’s HSD test at *p* = 0.05. Calibration curves used: apigenin-7-*O*-glucoside (*y* = 10683*x* – 45794; *R²* = 0.996; LOD = 0.10 µg/mL; LOQ = 0.53 µg/mL; peaks 1, 4 and 5) and quercetin-3-*O*-rutinoside (*y* = 13343*x* + 76751; *R²* = 0.9998; Level of detection (LOD) = 0.21 µg/mL; Level of quantification (LOQ) = 0.71 µg/mL; peaks 2, 3, 6 and 7).

**Table 3:** Antioxidant activities of the hydroethanolic extracts of the tested parsley leaves (mean ± SD, *n* = 3).

Cultivar Type	Cultivar Name	TBARS	OxHLIA (IC50, µg/mL)	
		(EC50, mg/mL)	Δt = 60 min	Δt = 120 min
Plain-leafed		1.6 ± 0.5 ^A^	112 ± 49 ^B^	204 ± 140 ^B^
	Festival 68	0.79 ± 0.03 ^c^	88 ± 2 ^d^	114 ± 2 ^c^ ^d^
	Astra	1.74 ± 0.08 ^b^	126 ± 2 ^b^	205 ± 3 ^b^
	Gigante Di Italia	1.98 ± 0.06 ^a^	106 ± 3 ^c^	150 ± 3 ^b^ ^c^
	Fest	1.93 ± 0.03 ^a^	48 ± 1 ^e^	90 ± 1 ^d^
	Rialto Bejo	1.78 ± 0.01 ^b^	190 ± 13 ^a^	462 ± 21 ^a^
	Festival 68	0.79 ± 0.03 ^c^	88 ± 2 ^d^	114 ± 2 ^c^ ^d^
	Astra	1.74 ± 0.08 ^b^	126 ± 2 ^b^	205 ± 3 ^b^
Curly-leafed		1.5 ± 0.6 ^A^	366 ± 91 ^A^	521 ± 95 ^A^
	Depuis 1743	1.82 ± 0.05 ^a^	325 ± 9 ^b^	507 ± 10 ^b^
	Moss Curled 2	1.9 ± 0.1 ^a^	288 ± 9 ^b^	421 ± 9 ^c^
	Mooskrause	0.77 ± 0.03 ^b^	484 ± 12 ^a^	636 ± 13 ^a^
Turnip-rooted		1.5 ± 0.3 ^A^	118 ± 66 ^B^	173 ± 96 ^B^
	Olomuńcka	0.99 ± 0.02 ^h^	175 ± 4 ^b^	239 ± 4 ^b^
	Pólna	1.99 ± 0.09 ^a^	101 ± 3 ^d^ ^e^ ^f^	150 ± 3 ^f^ ^g^
	Linga	1.62 ± 0.05 ^c^ ^d^	69 ± 2 ^h^	105 ± 2 ^i^
	Halblange Berlinska	1.51 ± 0.08 ^e^ ^f^	70 ± 2 ^h^	99 ± 2 ^i^
	Osborne	1.68 ± 0.09 ^c^	92 ± 2 ^f^ ^g^	146 ± 3 ^f^ ^g^
	Lenka	1.65 ± 0.05 ^c^ ^d^	78 ± 1 ^g^ ^h^	109 ± 2 ^i^
	Sonata	1.57 ± 0.08 ^d^ ^e^	98 ± 4 ^d^ ^e^ ^f^	158 ± 6 ^f^
	Kaśka	1.43 ± 0.07 ^f^	64 ± 2 ^h^	117 ± 2 ^h^ ^i^
	Vistula	0.82 ± 0.01 ^i^	78 ± 2 ^g^ ^h^	117 ± 2 ^h^ ^i^
	Konika	1.29 ± 0.07 ^g^	112 ± 4 ^d^	157 ± 6 ^f^
	Hanacka	1.42 ± 0.08 ^f^	109 ± 5 ^d^ ^e^	159 ± 8 ^e^ ^f^
	Halblange Eagle	1.43 ± 0.09 ^f^	107 ± 6 ^d^ ^e^ ^f^	145 ± 9 ^f^ ^g^
	Cukrowa	1.64 ± 0.06 ^c^ ^d^	138 ± 3 ^c^	179 ± 4 ^d^ ^e^
	Alba	1.26 ± 0.05 ^g^	96 ± 4 ^e^ ^f^	135 ± 4 ^g^ ^h^
	Arat	1.84 ± 0.08 ^b^	351 ± 15 ^a^	524 ± 18 ^a^
	Root parsley (common variety)	1.50 ± 0.03 ^e^ ^f^	139 ± 6 ^c^	213 ± 12 ^c^
	Berlinski Halblange Springer	1.86 ± 0.05 ^b^	130 ± 3 ^c^	187 ± 4 ^d^

Different capital Latin letters in the same column indicate significant differences between the means of the three types of cultivars according to Tukey’s honestly significant difference (HSD) test at *p* = 0.05, while different small Latin letters in the same column indicate significant differences between the means of the cultivars of the same type according to Tukey’s HSD test at *p* = 0.05.

**Table 4:** Antibacterial activity (minimal inhibition concentration (MIC) and minimal bactericidal concentration (MBC) mg/mL) of parsley leaves’ hydroethanolic extracts.

Plain-Leafed	Cultivar Name		*S. aureus* (ATCC 11632)	*B. cereus* (Clinical Isolate)	*L. monocytogenes* (NCTC 7973)	*E. coli* (ATCC 25922)	*S. Typhimurium* (ATCC 13311)	*En. cloacae* (ATCC 35030)
Plain-leafed	Festival 68	MIC	1.00	1.00	1.00	1.00	2.00	2.00
		MBC	2.00	2.00	2.00	2.00	4.00	4.00
	Astra	MIC	1.00	0.50	1.00	1.00	1.00	1.00
		MBC	2.00	1.00	2.00	2.00	2.00	2.00
	Gigante Di Italia	MIC	1.00	1.00	2.00	1.00	2.00	2.00
		MBC	2.00	2.00	4.00	2.00	4.00	4.00
	Fest	MIC	1.00	0.50	1.00	0.50	2.00	2.00
		MBC	2.00	1.00	2.00	1.00	4.00	4.00
	Rialto Bejo	MIC	4.00	1.00	1.00	0.50	1.00	2.00
		MBC	8.00	2.00	2.00	1.00	2.00	4.00
Curly-leafed	Depuis 1743	MIC	2.00	0.50	1.00	1.00	1.00	1.00
		MBC	4.00	1.00	2.00	2.00	2.00	2.00
	Moss Curled 2	MIC	2.00	1.00	1.00	1.00	1.00	1.00
		MBC	4.00	2.00	2.00	2.00	2.00	2.00
	Mooskrause	MIC	4.00	1.00	2.00	1.00	1.00	2.00
		MBC	8.00	2.00	4.00	2.00	2.00	4.00
Turnip-rooted	Olomuńcka	MIC	1.00	0.50	1.00	1.00	2.00	1.00
		MBC	2.00	1.00	2.00	2.00	4.00	2.00
	Pólna	MIC	1.00	1.00	1.00	1.00	2.00	2.00
		MBC	2.00	2.00	2.00	2.00	4.00	4.00
	Linga	MIC	4.00	1.00	1.00	0.50	1.00	1.00
		MBC	8.00	2.00	2.00	1.00	2.00	2.00
	Halblange Berlinska	MIC	4.00	4.00	4.00	4.00	4.00	4.00
		MBC	8.00	8.00	8.00	8.00	8.00	8.00
	Osborne	MIC	4.00	2.00	2.00	1.00	2.00	4.00
		MBC	8.00	4.00	4.00	2.00	4.00	8.00
	Lenka	MIC	1.00	1.00	1.00	0.50	1.00	2.00
		MBC	2.00	2.00	2.00	1.00	2.00	4.00
	Sonata	MIC	2.00	0.50	1.00	0.50	1.00	1.00
		MBC	4.00	1.00	2.00	1.00	2.00	2.00
	Kaśka	MIC	4.00	1.00	1.00	0.50	1.00	2.00
		MBC	8.00	2.00	2.00	1.00	2.00	4.00
	Vistula	MIC	1.00	1.00	1.00	0.50	1.00	1.00
		MBC	2.00	2.00	2.00	1.00	2.00	2.00
	Konika	MIC	2.00	2.00	1.00	1.00	1.00	4.00
		MBC	4.00	4.00	2.00	2.00	2.00	8.00
	Hanacka	MIC	2.00	1.00	1.00	2.00	1.00	2.00
		MBC	4.00	2.00	2.00	4.00	2.00	4.00
	Halblange Eagle	MIC	2.00	1.00	1.00	1.00	1.00	1.00
		MBC	4.00	2.00	2.00	2.00	2.00	2.00
	Cukrowa	MIC	1.00	1.00	1.00	1.00	2.00	2.00
		MBC	2.00	2.00	2.00	2.00	4.00	4.00
	Alba	MIC	1.00	1.00	1.00	1.00	1.00	1.00
		MBC	2.00	2.00	2.00	2.00	2.00	2.00
	Arat	MIC	4.00	2.00	2.00	2.00	2.00	2.00
		MBC	8.00	4.00	4.00	4.00	4.00	4.00
	Root parsley (common variety)	MIC	2.00	2.00	1.00	1.00	1.00	1.00
		MBC	4.00	4.00	2.00	2.00	2.00	2.00
	Berlinski Halblange Springer	MIC	2.00	1.00	1.00	0.50	1.00	1.00
		MBC	4.00	2.00	2.00	1.00	2.00	2.00
Positive controls	E211	MIC	4.00	0.50	1.00	1.00	1.00	2.00
		MBC	4.00	0.50	2.00	2.00	2.00	4.00
	E224	MIC	1.0	2.0	0.5	0.5	1.0	0.5
		MBC	1.0	4.0	1.0	1.0	1.0	0.5

**Table 5:** Antifungal activity of parsley leaves’ hydroethanolic extracts (MIC and minimal fungicidal concentration (MFC) mg/mL).

Plain-Leafed	Cultivar Name		*A. ochraceus* (ATCC 12066)	*A. versicolor* (ATCC 11730)	*A. fumigatus* (ATCC 9197)	*P. funiculosum* (ATCC 36839)	*P. verrucosum* var. *cyclopium* (Food Isolate)	*T. viride* (IAM 5061)
Plain-leafed	Festival 68	MIC	1.00	0.50	0.50	0.50	0.50	1.00
		MFC	2.00	1.00	1.00	1.00	1.00	2.00
	Astra	MIC	1.00	0.50	0.50	0.50	0.50	1.00
		MFC	2.00	1.00	1.00	1.00	1.00	2.00
	Gigante Di Italia	MIC	2.00	2.00	2.00	1.00	1.00	1.00
		MFC	4.00	4.00	4.00	2.00	2.00	2.00
	Fest	MIC	4.00	2.00	4.00	1.00	2.00	1.00
		MFC	8.00	4.00	8.00	2.00	4.00	2.00
	Rialto Bejo	MIC	0.50	0.50	0.25	0.50	0.50	0.25
		MFC	1.00	1.00	0.50	1.00	1.00	0.50
Curly-leafed	Depuis 1743	MIC	2.00	1.00	2.00	4.00	1.00	1.00
		MFC	4.00	2.00	4.00	8.00	2.00	2.00
	Moss Curled 2	MIC	4.00	2.00	4.00	2.00	2.00	1.00
		MFC	8.00	4.00	8.00	4.00	4.00	2.00
	Mooskrause	MIC	0.50	0.50	0.25	0.50	0.50	0.25
		MFC	1.00	1.00	0.50	1.00	1.00	0.50
Turnip-rooted	Olomuńcka	MIC	2.00	1.00	2.00	4.00	4.00	1.00
		MFC	4.00	2.00	4.00	8.00	8.00	2.00
	Pólna	MIC	4.00	2.00	2.00	2.00	1.00	1.00
		MFC	8.00	4.00	4.00	4.00	2.00	2.00
	Linga	MIC	1.00	2.00	4.00	4.00	2.00	0.50
		MFC	2.00	4.00	8.00	8.00	4.00	1.00
	Halblange Berlinska	MIC	1.00	4.00	2.00	2.00	2.00	0.50
		MFC	2.00	8.00	4.00	4.00	4.00	1.00
	Osborne	MIC	2.00	4.00	2.00	1.00	1.00	2.00
		MFC	4.00	8.00	4.00	2.00	2.00	4.00
	Lenka	MIC	2.00	2.00	1.00	1.00	2.00	1.00
		MFC	4.00	4.00	2.00	2.00	4.00	2.00
	Sonata	MIC	2.00	4.00	2.00	4.00	2.00	1.00
		MFC	4.00	8.00	4.00	8.00	4.00	2.00
	Kaśka	MIC	4.00	4.00	4.00	2.00	2.00	0.50
		MFC	8.00	8.00	8.00	4.00	4.00	1.00
	Vistula	MIC	2.00	4.00	1.00	2.00	4.00	1.00
		MFC	4.00	8.00	2.00	4.00	8.00	2.00
	Konika	MIC	1.00	2.00	4.00	4.00	2.00	1.00
		MFC	2.00	4.00	8.00	8.00	4.00	2.00
	Hanacka	MIC	0.50	0.50	0.50	0.50	0.50	0.25
		MFC	1.00	1.00	1.00	1.00	1.00	0.50
	Halblange Eagle	MIC	0.50	0.50	0.25	0.50	0.25	0.25
		MFC	1.00	1.00	0.50	1.00	0.50	0.50
	Cukrowa	MIC	0.50	0.50	0.25	0.50	0.25	0.25
		MFC	1.00	1.00	0.50	1.00	0.50	0.50
	Alba	MIC	1.00	1.00	1.00	1.00	0.50	0.50
		MFC	2.00	2.00	2.00	2.00	1.00	1.00
	Arat	MIC	1.00	1.00	0.50	0.50	0.50	0.50
		MFC	2.00	2.00	1.00	1.00	1.00	1.00
	Root parsley (common variety)	MIC	0.50	0.50	0.25	0.50	0.50	0.25
		MFC	1.00	1.00	0.50	1.00	1.00	0.50
	Berlinski Halblange Springer	MIC	0.50	0.50	0.50	0.50	0.50	0.50
		MFC	1.00	1.00	1.00	1.00	1.00	1.00
Positive controls	E211	MIC	1.00	2.00	1.00	1.00	2.00	1.00
		MFC	2.00	4.00	2.00	2.00	4.00	2.00
	E224	MIC	1.00	1.00	1.00	0.50	1.00	0.50
		MFC	1.00	1.00	1.00	0.50	1.00	0.50

**Table 6:** List of cultivars used in the present experiment (type and name of cultivar and seed company).

Cultivar Type	Cultivar Name	Seed Company	Country of Origin
Plain-leafed			
	Festival 68	W. Legutko	Poland
	Astra	Polan	Poland
	Gigante Di Italia	W. Legutko	Poland
	Fest	Polan	Poland
	Rialto Bejo	Bejo Zaden	The Netherlands
Curly-leafed			
	Depuis 1743	Vilmorin Garden	Poland
	Moss Curled 2	W. Legutko	Poland
	Mooskrause	Semenarna Ljubljana	Slovenia
Turnip-rooted			
	Olomuńcka	W. Legutko	Poland
	Pólna	Toraf	Poland
	Linga	Polan	Poland
	Halblange Berlinska	W. Legutko	Poland
	Osborne	PNOS	Poland
	Lenka	W. Legutko	Poland
	Sonata	PNOS	Poland
	Kaśka	PNOS	Poland
	Vistula	Polan	Poland
	Konika	Toraf	Poland
	Hanacka	Vilmorin Garden	Poland
	Halblange Eagle	W. Legutko	Poland
	Cukrowa	W. Legutko	Poland
	Alba	Vilmorin Garden	Poland
	Arat	Bejo Zaden	The Netherlands
	Root parsley (common variety)	-	-
	Berlinski Halblange Springer	Springer Semena	Poland
